# Poly[[aqua­tri-μ_3_-hydroxido-(μ_4_-2-phos­phon­ato­ethane­sulfonato)­dierbium(III)] monohydrate]

**DOI:** 10.1107/S1600536808033230

**Published:** 2008-10-18

**Authors:** Andreas Sonnauer, Norbert Stock

**Affiliations:** a Institute of Inorganic Chemistry, Christian-Albrechts-University, Max-Eyth-Strasse 2, D 24118 Kiel, Germany

## Abstract

The crystal structure of the title compound, {[Er_2_(C_2_H_4_O_6_PS)(OH)_3_(H_2_O)]·H_2_O}_*n*_, consists of two Er^3+^ ions, one (C_2_H_4_O_6_PS)^3−^ ion, three OH^−^ ions, and two water mol­ecule. The Er^3+^ ions form ErO_8_ polyhedra., which are connected by μ- and μ_3_-O atoms. Thus, inorganic Er–O–Er layers of edge- and face-sharing polyhedra are observed. Whereas most often in metal phosphono­sulfonates the organic linker bridges adjacent layers, in the title compound, the (O_3_PC_2_H_4_SO_3_)^3−^ anion is only connected to one Er–O–Er layer. Short interatomic O⋯O distances [2.898 (8), 2.997 (14) and 2.768 (10) Å] indicate hydrogen bonding between the layers. The noncoordinated water mol­ecules are located between the layers.

## Related literature

For related structures, see: Sonnauer *et al.* (2007 ▶); Sonnauer & Stock (2008*a* ▶,*b* ▶); Benedetto *et al.* (1997 ▶); Adani *et al.* (1998 ▶); Du *et al.* (2006*a* ▶,*b* ▶); Du, Li *et al.* (2007 ▶); Du, Prosvirin & Mao (2007 ▶); Du, Xu *et al.* (2007 ▶).
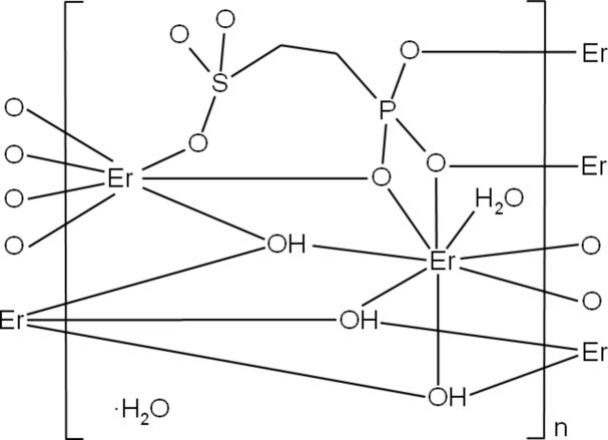


## Experimental

### 

#### Crystal data

[Er_2_(C_2_H_4_O_6_PS)(OH)_3_(H_2_O)]·H_2_O
*M* *_r_* = 608.65
Triclinic, 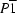

*a* = 5.8621 (6) Å
*b* = 9.0443 (9) Å
*c* = 11.6240 (11) Å
α = 105.543 (12)°
β = 101.713 (11)°
γ = 101.885 (11)°
*V* = 558.81 (10) Å^3^
*Z* = 2
Mo *K*α radiation
μ = 15.29 mm^−1^
*T* = 293 (2) K
0.08 × 0.07 × 0.06 mm

#### Data collection

Stoe IPDS-1 diffractometer
Absorption correction: ψ scan (*X-RED* and *X-SHAPE*; Stoe & Cie, 1999 ▶) *T*_min_ = 0.293, *T*_max_ = 0.399
4731 measured reflections
2664 independent reflections
2207 reflections with *I* > 2σ(*I*)
*R*_int_ = 0.041

#### Refinement

*R*[*F* ^2^ > 2σ(*F* ^2^)] = 0.031
*wR*(*F* ^2^) = 0.082
*S* = 0.97
2664 reflections
155 parameters
H-atom parameters constrained
Δρ_max_ = 1.61 e Å^−3^
Δρ_min_ = −2.07 e Å^−3^

### 

Data collection: *IPDS Program Package* (Stoe & Cie, 1998 ▶); cell refinement: *IPDS Program Package*; data reduction: *IPDS Program Package*; program(s) used to solve structure: *SHELXS97* (Sheldrick, 2008 ▶); program(s) used to refine structure: *SHELXL97* (Sheldrick, 2008 ▶); molecular graphics: *DIAMOND* (Brandenburg & Putz, 1999 ▶); software used to prepare material for publication: *publCIF* (Westrip, 2008 ▶).

## Supplementary Material

Crystal structure: contains datablocks I, global. DOI: 10.1107/S1600536808033230/bt2804sup1.cif

Structure factors: contains datablocks I. DOI: 10.1107/S1600536808033230/bt2804Isup2.hkl

Additional supplementary materials:  crystallographic information; 3D view; checkCIF report

## Figures and Tables

**Fig. 1.**
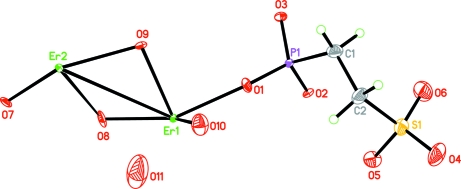
Asymmetric unit of the title compound. Displacement ellipsoides are drawn at 50% propability level.

**Fig. 2.**
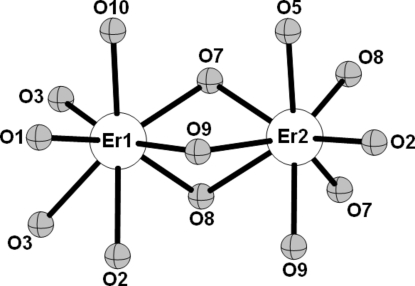
First coordination sphere of the erbium ions.

**Fig. 3.**
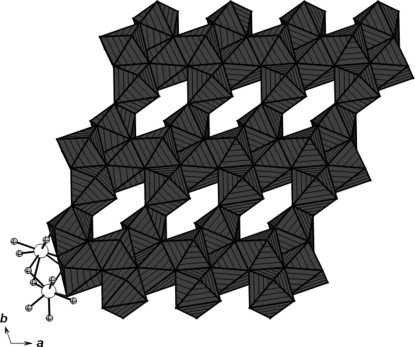
Inorganic Er–O–Er layer in the ab-plane of edge- and face-sharing ErO8 polyhedra.

**Fig. 4.**
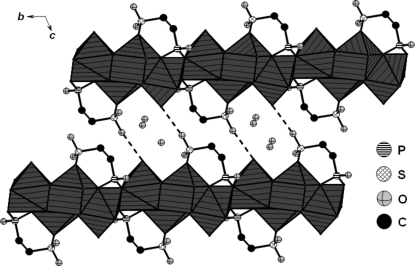
The anions (O3PC2H4SO3)3- are connected to one Er–O–Er layer at a time. The layers are linked via hydrogen bonds between the sulfonate groups and the coordinating water molecules into a three-dimensional structure.

## supplementary crystallographic information

### Comment

Inorganic–organic hybrid materials based on metal carboxylates, sulfonates and phosphonates are intensively investigated due to their potential application *i.e.* in the field of gas separation, storage, as well as catalysis, or as sensor materials. We are interested in the use of organic ligands containing two or more different functional groups for the synthesis of functionalized hybrid compounds. Although a large number of metal phosphonates and metal sulfonates have been reported in the literature, compounds based on ligands containing simultaneously a phosphonic as well as a sulfonic acid group have only recently been investigated. These few studies are limited to the use of linker molecules based on rigid phosphonoarylsulfonic acids (Benedetto *et al.*, 1997; Adani *et al.*, 1998; Du *et al.*,  2006*a*,*b*; Du, Li *et al.*, 2007; Du, Prosvirin & Mao, 2007; Du, Xu *et al.*, 2007). Our group has started a systematic investigation using the flexible linker 2-phosphonoethansulfonic acid, which has been recently reported in the literature (Sonnauer *et al.*, 2007; Sonnauer & Stock, 2008*a*,*b*). Here we describe the crystal structure of the new rare earth phosphonatosulfonate [Er_2_(O_3_PC_2_H_4_SO_3_)(OH)_3_(H_2_O)]H_2_O, which was obtained during a high-throughput screening experiment.

The title compound consists of two crystallographic independent erbium ions, one fully deprotonated (O_3_PC_2_H_4_SO_3_)^3-^ anion, three hydroxide ions, as well as two water molecules (one is coordinated to a erbium ion) (Fig. 1). The erbium ions are coordinated eightfold by oxygen atoms and form ErO_8_ polyhedra (Fig. 2), which are linked (edge- and face-sharing) to inorganic layers Er–O–Er in the *ab*-plane (Fig. 3). While most often in metal phosphonosulfonates the organic linker bridges adjacent layers, in [Er_2_(O_3_PC_2_H_4_SO_3_)(OH)_3_(H_2_O)]H_2_O the anion (O_3_PC_2_H_4_SO_3_)^3-^ is only connected to one Er–O–Er layer. These layers are further linked *via* hydrogen bonds into a three-dimensional structure (Fig. 4). The non-coordinating H_2_O molecules are located in between the layers.

### Experimental

H_2_O_3_PC_2_H_4_SO_3_H was synthesized as previously reported (Sonnauer & Stock, 2008*b*). All other reagents were of analytical grade (Aldrich and Fluka) and were used without furhter purification. For the synthesis a special high-throughput reactor system was used. 79 µl (0.016 mmol) of 0.2 *M* Er(CH_3_CO_2_)_3_.4H_2_O, 53.2 µl (0.032 mmol) of 0.5 *M* H_2_O_3_PC_2_H_4_SO_3_H, 23.7 µl (0.048 mmol) of 2.0 *M* NaOH, and 54 µl H_2_O were filled in a teflon reactor and heated to 160 °C for 48 h. After filtration the pink rod-shaped single crystals were isolated.

### Refinement

The H atoms connected to C atoms were positioned with idealized geometry and were refined isotropically with *U_eq_*(H) = 1.2 *U_eq_*(C) of the parent atom using a riding model with C—H = 0.97 Å. The H atoms connected to O atoms could not be located from the difference Fourier map and were omitted from refinement.

### Crystal data

**Table:**

[Er_2_(C_2_H_4_O_6_PS)(OH)_3_(H_2_O)]·H_2_O	*Z* = 2
*M**_r_* = 608.65	*F*(000) = 554
Triclinic, *P*1	*D*_x_ = 3.611 Mg m^−^^3^
Hall symbol: -P 1	Mo *K*α radiation, λ = 0.71073 Å
*a* = 5.8621 (6) Å	Cell parameters from 4793 reflections
*b* = 9.0443 (9) Å	θ = 2.5–28°
*c* = 11.6240 (11) Å	µ = 15.29 mm^−^^1^
α = 105.543 (12)°	*T* = 293 K
β = 101.713 (11)°	Rod, pink
γ = 101.885 (11)°	0.08 × 0.07 × 0.06 mm
*V* = 558.81 (10) Å^3^	

### Data collection

**Table:**

Stoe IPDS-1 diffractometer	2664 independent reflections
Radiation source: fine-focus sealed tube	2207 reflections with *I* > 2σ(*I*)
graphite	*R*_int_ = 0.041
φ scans	θ_max_ = 28.1°, θ_min_ = 2.4°
Absorption correction: ψ scan (X-RED and X-SHAPE; Stoe & Cie, 1999)	*h* = −7→7
*T*_min_ = 0.293, *T*_max_ = 0.399	*k* = −11→11
4731 measured reflections	*l* = −15→15

### Refinement

**Table:**

Refinement on *F*^2^	Secondary atom site location: difference Fourier map
Least-squares matrix: full	Hydrogen site location: inferred from neighbouring sites
*R*[*F*^2^ > 2σ(*F*^2^)] = 0.031	H-atom parameters constrained
*wR*(*F*^2^) = 0.082	*w* = 1/[σ^2^(*F*_o_^2^) + (0.0577*P*)^2^] where *P* = (*F*_o_^2^ + 2*F*_c_^2^)/3
*S* = 0.97	(Δ/σ)_max_ = 0.001
2664 reflections	Δρ_max_ = 1.61 e Å^−^^3^
155 parameters	Δρ_min_ = −2.07 e Å^−^^3^
0 restraints	Extinction correction: SHELXL97 (Sheldrick, 2008), Fc^*^=kFc[1+0.001xFc^2^λ^3^/sin(2θ)]^-1/4^
Primary atom site location: structure-invariant direct methods	Extinction coefficient: 0.0044 (5)

### Special details

**Table:**

Geometry. All e.s.d.'s (except the e.s.d. in the dihedral angle between two l.s. planes) are estimated using the full covariance matrix. The cell e.s.d.'s are taken into account individually in the estimation of e.s.d.'s in distances, angles and torsion angles; correlations between e.s.d.'s in cell parameters are only used when they are defined by crystal symmetry. An approximate (isotropic) treatment of cell e.s.d.'s is used for estimating e.s.d.'s involving l.s. planes.
Refinement. Refinement of *F*^2^ against ALL reflections. The weighted *R*-factor *wR* and goodness of fit *S* are based on *F*^2^, conventional *R*-factors *R* are based on *F*, with *F* set to zero for negative *F*^2^. The threshold expression of *F*^2^ > σ(*F*^2^) is used only for calculating *R*-factors(gt) *etc*. and is not relevant to the choice of reflections for refinement. *R*-factors based on *F*^2^ are statistically about twice as large as those based on *F*, and *R*- factors based on ALL data will be even larger.

### Fractional atomic coordinates and isotropic or equivalent isotropic displacement parameters (Å^2^)

**Table:**

	*x*	*y*	*z*	*U*_iso_*/*U*_eq_	
Er1	0.21965 (5)	0.71416 (3)	0.57132 (3)	0.00796 (12)	
Er2	0.27992 (5)	1.10732 (3)	0.55352 (3)	0.00839 (12)	
S1	0.6239 (4)	0.2840 (2)	0.86984 (18)	0.0197 (4)	
P1	0.6867 (3)	0.5153 (2)	0.65414 (16)	0.0080 (3)	
O1	0.5161 (10)	0.6197 (6)	0.6590 (5)	0.0150 (10)	
O2	0.5556 (9)	0.3377 (5)	0.5872 (5)	0.0106 (9)	
O3	0.8809 (9)	0.5494 (6)	0.5852 (5)	0.0129 (10)	
O4	0.5879 (13)	0.2641 (8)	0.9845 (6)	0.0324 (15)	
O5	0.3963 (11)	0.2164 (7)	0.7714 (5)	0.0214 (12)	
O6	0.8156 (13)	0.2224 (8)	0.8314 (7)	0.0323 (15)	
O7	−0.0251 (9)	1.0829 (6)	0.3821 (5)	0.0110 (9)	
O8	0.0459 (10)	0.7988 (6)	0.4152 (5)	0.0170 (11)	
O9	0.5197 (9)	0.9454 (5)	0.6002 (5)	0.0100 (9)	
O10	0.3173 (12)	0.8253 (7)	0.7926 (6)	0.0255 (13)	
C1	0.8513 (15)	0.5462 (9)	0.8095 (7)	0.0185 (15)	
H1A	0.9760	0.4907	0.8057	0.022*	
H1B	0.9328	0.6592	0.8479	0.022*	
C2	0.7080 (16)	0.4931 (10)	0.8961 (7)	0.0217 (16)	
H2A	0.5621	0.5287	0.8864	0.026*	
H2B	0.8053	0.5464	0.9813	0.026*	
O11	0.093 (2)	1.0351 (13)	0.9079 (12)	0.078 (4)	

### Atomic displacement parameters (Å^2^)

**Table:**

	*U*^11^	*U*^22^	*U*^33^	*U*^12^	*U*^13^	*U*^23^
Er1	0.00654 (18)	0.00560 (17)	0.01466 (18)	0.00295 (11)	0.00471 (12)	0.00566 (12)
Er2	0.00577 (18)	0.00797 (17)	0.01466 (18)	0.00329 (12)	0.00454 (12)	0.00646 (12)
S1	0.0221 (10)	0.0209 (9)	0.0176 (9)	0.0061 (8)	0.0051 (8)	0.0086 (7)
P1	0.0070 (8)	0.0064 (7)	0.0131 (8)	0.0029 (6)	0.0043 (6)	0.0053 (6)
O1	0.015 (3)	0.015 (2)	0.022 (3)	0.012 (2)	0.008 (2)	0.009 (2)
O2	0.010 (2)	0.009 (2)	0.015 (2)	0.0000 (18)	0.0045 (19)	0.0069 (18)
O3	0.010 (3)	0.017 (2)	0.016 (2)	0.004 (2)	0.008 (2)	0.009 (2)
O4	0.043 (4)	0.037 (4)	0.018 (3)	0.006 (3)	0.006 (3)	0.016 (3)
O5	0.019 (3)	0.025 (3)	0.012 (2)	0.000 (2)	−0.001 (2)	0.001 (2)
O6	0.032 (4)	0.035 (4)	0.039 (4)	0.019 (3)	0.015 (3)	0.014 (3)
O7	0.004 (2)	0.012 (2)	0.016 (2)	0.0030 (18)	0.0005 (19)	0.0030 (18)
O8	0.020 (3)	0.024 (3)	0.018 (3)	0.019 (2)	0.009 (2)	0.012 (2)
O9	0.007 (2)	0.009 (2)	0.015 (2)	0.0002 (18)	0.0050 (19)	0.0058 (18)
O10	0.032 (4)	0.027 (3)	0.022 (3)	0.015 (3)	0.009 (3)	0.009 (2)
C1	0.015 (4)	0.018 (4)	0.020 (4)	−0.001 (3)	0.003 (3)	0.006 (3)
C2	0.023 (4)	0.025 (4)	0.018 (4)	0.003 (3)	0.006 (3)	0.012 (3)
O11	0.108 (9)	0.093 (7)	0.119 (9)	0.082 (7)	0.093 (8)	0.086 (7)

### Geometric parameters (Å, °)

**Table:**

Er1—O1	2.269 (5)	S1—O4	1.442 (6)
Er1—O3^i^	2.287 (5)	S1—O6	1.449 (7)
Er1—O8	2.287 (5)	S1—O5	1.460 (6)
Er1—O9	2.334 (5)	S1—C2	1.777 (8)
Er1—O7^ii^	2.354 (5)	P1—O1	1.509 (5)
Er1—O10	2.394 (6)	P1—O2	1.535 (5)
Er1—O3^iii^	2.450 (5)	P1—O3	1.547 (5)
Er1—O2^iii^	2.475 (5)	P1—C1	1.784 (8)
Er1—P1^iii^	3.1010 (18)	P1—Er1^iii^	3.1010 (18)
Er1—Er2	3.5662 (5)	O2—Er2^vi^	2.242 (4)
Er1—Er2^iv^	3.7884 (6)	O2—Er1^iii^	2.475 (5)
Er1—Er2^ii^	3.8428 (6)	O3—Er1^vii^	2.287 (5)
Er2—O2^v^	2.242 (5)	O3—Er1^iii^	2.450 (5)
Er2—O8^ii^	2.299 (5)	O5—Er2^vi^	2.353 (5)
Er2—O7	2.317 (5)	O7—Er1^ii^	2.354 (5)
Er2—O9	2.319 (5)	O7—Er2^ii^	2.416 (5)
Er2—O9^iv^	2.329 (5)	O8—Er2^ii^	2.299 (5)
Er2—O5^v^	2.353 (5)	O9—Er2^iv^	2.329 (5)
Er2—O7^ii^	2.416 (5)	C1—C2	1.544 (10)
Er2—O8	2.716 (6)	C1—H1A	0.9700
Er2—Er2^ii^	3.2420 (8)	C1—H1B	0.9700
Er2—Er2^iv^	3.7479 (7)	C2—H2A	0.9700
Er2—Er1^iv^	3.7884 (6)	C2—H2B	0.9700
			
O1—Er1—O3^i^	101.48 (18)	O7—Er2—O8	67.01 (16)
O1—Er1—O8	150.13 (18)	O9—Er2—O8	70.88 (16)
O3^i^—Er1—O8	99.53 (19)	O9^iv^—Er2—O8	76.51 (16)
O1—Er1—O9	87.87 (18)	O5^v^—Er2—O8	126.96 (17)
O3^i^—Er1—O9	160.71 (18)	O7^ii^—Er2—O8	54.15 (16)
O8—Er1—O9	78.90 (19)	O2^v^—Er2—Er2^ii^	148.76 (13)
O1—Er1—O7^ii^	141.65 (18)	O8^ii^—Er2—Er2^ii^	55.60 (15)
O3^i^—Er1—O7^ii^	85.63 (17)	O7—Er2—Er2^ii^	48.05 (12)
O8—Er1—O7^ii^	60.84 (17)	O9—Er2—Er2^ii^	108.32 (12)
O9—Er1—O7^ii^	76.79 (17)	O9^iv^—Er2—Er2^ii^	109.66 (12)
O1—Er1—O10	70.82 (19)	O5^v^—Er2—Er2^ii^	112.02 (14)
O3^i^—Er1—O10	86.1 (2)	O7^ii^—Er2—Er2^ii^	45.52 (12)
O8—Er1—O10	131.87 (19)	O8—Er2—Er2^ii^	44.30 (11)
O9—Er1—O10	81.02 (19)	O2^v^—Er2—Er1	142.54 (13)
O7^ii^—Er1—O10	72.17 (18)	O8^ii^—Er2—Er1	112.98 (13)
O1—Er1—O3^iii^	80.62 (18)	O7—Er2—Er1	105.98 (12)
O3^i^—Er1—O3^iii^	69.78 (19)	O9—Er2—Er1	40.13 (11)
O8—Er1—O3^iii^	87.03 (18)	O9^iv^—Er2—Er1	90.81 (11)
O9—Er1—O3^iii^	128.90 (16)	O5^v^—Er2—Er1	90.97 (14)
O7^ii^—Er1—O3^iii^	135.88 (18)	O7^ii^—Er2—Er1	40.96 (11)
O10—Er1—O3^iii^	137.99 (18)	O8—Er2—Er1	39.89 (10)
O1—Er1—O2^iii^	76.65 (17)	Er2^ii^—Er2—Er1	68.536 (15)
O3^i^—Er1—O2^iii^	128.23 (17)	O2^v^—Er2—Er2^iv^	88.10 (13)
O8—Er1—O2^iii^	73.69 (17)	O8^ii^—Er2—Er2^iv^	168.52 (15)
O9—Er1—O2^iii^	70.13 (16)	O7—Er2—Er2^iv^	108.96 (12)
O7^ii^—Er1—O2^iii^	127.72 (16)	O9—Er2—Er2^iv^	36.36 (11)
O10—Er1—O2^iii^	136.9 (2)	O9^iv^—Er2—Er2^iv^	36.18 (12)
O3^iii^—Er1—O2^iii^	58.77 (15)	O5^v^—Er2—Er2^iv^	110.34 (15)
O1—Er1—P1^iii^	76.23 (14)	O7^ii^—Er2—Er2^iv^	103.29 (11)
O3^i^—Er1—P1^iii^	99.19 (13)	O8—Er2—Er2^iv^	69.65 (11)
O8—Er1—P1^iii^	79.66 (13)	Er2^ii^—Er2—Er2^iv^	113.801 (19)
O9—Er1—P1^iii^	99.42 (12)	Er1—Er2—Er2^iv^	62.333 (12)
O7^ii^—Er1—P1^iii^	140.41 (12)	O2^v^—Er2—Er1^iv^	38.73 (13)
O10—Er1—P1^iii^	147.01 (15)	O8^ii^—Er2—Er1^iv^	127.02 (12)
O3^iii^—Er1—P1^iii^	29.49 (11)	O7—Er2—Er1^iv^	93.57 (12)
O2^iii^—Er1—P1^iii^	29.30 (11)	O9—Er2—Er1^iv^	85.59 (11)
O1—Er1—Er2	126.95 (13)	O9^iv^—Er2—Er1^iv^	35.72 (12)
O3^i^—Er1—Er2	126.08 (13)	O5^v^—Er2—Er1^iv^	109.15 (14)
O8—Er1—Er2	49.59 (14)	O7^ii^—Er2—Er1^iv^	159.77 (11)
O9—Er1—Er2	39.82 (12)	O8—Er2—Er1^iv^	112.23 (11)
O7^ii^—Er1—Er2	42.28 (11)	Er2^ii^—Er2—Er1^iv^	138.048 (15)
O10—Er1—Er2	88.68 (14)	Er1—Er2—Er1^iv^	118.815 (12)
O3^iii^—Er1—Er2	133.33 (11)	Er2^iv^—Er2—Er1^iv^	56.483 (10)
O2^iii^—Er1—Er2	88.92 (10)	O4—S1—O6	114.3 (4)
P1^iii^—Er1—Er2	112.89 (3)	O4—S1—O5	110.4 (4)
O1—Er1—Er2^iv^	81.22 (13)	O6—S1—O5	111.1 (4)
O3^i^—Er1—Er2^iv^	161.90 (13)	O4—S1—C2	106.3 (4)
O8—Er1—Er2^iv^	72.40 (14)	O6—S1—C2	107.4 (4)
O9—Er1—Er2^iv^	35.63 (12)	O5—S1—C2	106.9 (4)
O7^ii^—Er1—Er2^iv^	103.46 (11)	O1—P1—O2	112.9 (3)
O10—Er1—Er2^iv^	111.42 (16)	O1—P1—O3	116.1 (3)
O3^iii^—Er1—Er2^iv^	93.28 (11)	O2—P1—O3	103.3 (3)
O2^iii^—Er1—Er2^iv^	34.51 (11)	O1—P1—C1	108.1 (4)
P1^iii^—Er1—Er2^iv^	63.81 (3)	O2—P1—C1	110.6 (3)
Er2—Er1—Er2^iv^	61.185 (12)	O3—P1—C1	105.5 (3)
O1—Er1—Er2^ii^	175.89 (14)	O1—P1—Er1^iii^	130.4 (2)
O3^i^—Er1—Er2^ii^	78.19 (13)	O2—P1—Er1^iii^	52.12 (18)
O8—Er1—Er2^ii^	33.17 (13)	O3—P1—Er1^iii^	51.2 (2)
O9—Er1—Er2^ii^	91.27 (12)	C1—P1—Er1^iii^	121.5 (3)
O7^ii^—Er1—Er2^ii^	34.35 (12)	P1—O1—Er1	153.3 (3)
O10—Er1—Er2^ii^	105.08 (14)	P1—O2—Er2^vi^	154.7 (3)
O3^iii^—Er1—Er2^ii^	103.01 (12)	P1—O2—Er1^iii^	98.6 (2)
O2^iii^—Er1—Er2^ii^	106.83 (11)	Er2^vi^—O2—Er1^iii^	106.76 (19)
P1^iii^—Er1—Er2^ii^	107.88 (4)	P1—O3—Er1^vii^	150.2 (3)
Er2—Er1—Er2^ii^	51.734 (13)	P1—O3—Er1^iii^	99.3 (2)
Er2^iv^—Er1—Er2^ii^	100.378 (12)	Er1^vii^—O3—Er1^iii^	110.22 (19)
O2^v^—Er2—O8^ii^	100.04 (19)	S1—O5—Er2^vi^	136.5 (3)
O2^v^—Er2—O7	105.14 (17)	Er2—O7—Er1^ii^	110.7 (2)
O8^ii^—Er2—O7	61.23 (18)	Er2—O7—Er2^ii^	86.43 (16)
O2^v^—Er2—O9	102.46 (17)	Er1^ii^—O7—Er2^ii^	96.76 (17)
O8^ii^—Er2—O9	146.13 (16)	Er1—O8—Er2^ii^	113.9 (2)
O7—Er2—O9	133.96 (17)	Er1—O8—Er2	90.51 (19)
O2^v^—Er2—O9^iv^	74.44 (17)	Er2^ii^—O8—Er2	80.09 (15)
O8^ii^—Er2—O9^iv^	138.60 (17)	Er2—O9—Er2^iv^	107.46 (18)
O7—Er2—O9^iv^	80.31 (17)	Er2—O9—Er1	100.05 (18)
O9—Er2—O9^iv^	72.54 (18)	Er2^iv^—O9—Er1	108.64 (19)
O2^v^—Er2—O5^v^	77.68 (19)	C2—C1—P1	117.8 (6)
O8^ii^—Er2—O5^v^	79.5 (2)	C2—C1—H1A	107.9
O7—Er2—O5^v^	140.67 (19)	P1—C1—H1A	107.9
O9—Er2—O5^v^	80.92 (19)	C2—C1—H1B	107.9
O9^iv^—Er2—O5^v^	135.8 (2)	P1—C1—H1B	107.9
O2^v^—Er2—O7^ii^	153.74 (18)	H1A—C1—H1B	107.2
O8^ii^—Er2—O7^ii^	72.76 (17)	C1—C2—S1	115.0 (6)
O7—Er2—O7^ii^	93.57 (16)	C1—C2—H2A	108.5
O9—Er2—O7^ii^	75.87 (16)	S1—C2—H2A	108.5
O9^iv^—Er2—O7^ii^	127.80 (16)	C1—C2—H2B	108.5
O5^v^—Er2—O7^ii^	76.18 (18)	S1—C2—H2B	108.5
O2^v^—Er2—O8	150.83 (16)	H2A—C2—H2B	107.5
O8^ii^—Er2—O8	99.91 (16)		

Symmetry codes: (i) *x*−1, *y*, *z*; (ii) −*x*, −*y*+2, −*z*+1; (iii) −*x*+1, −*y*+1, −*z*+1; (iv) −*x*+1, −*y*+2, −*z*+1; (v) *x*, *y*+1, *z*; (vi) *x*, *y*−1, *z*; (vii) *x*+1, *y*, *z*.
